# Perfusion index variations in clinically and hemodynamically stable preterm newborns in the first week of life

**DOI:** 10.1186/1824-7288-36-6

**Published:** 2010-01-18

**Authors:** Francesco Cresi, Emanuela Pelle, Roberto Calabrese, Luciana Costa, Daniela Farinasso, Leandra Silvestro

**Affiliations:** 1 Neonatal Care Unit, Regina Margherita Children's Hospital, Department of Pediatrics, University of Turin, Italy; 2 Department of Pediatrics, University of Turin, Italy

## Abstract

**Background:**

The perfusion index, derived from the pulse oximeter signal, seems to be an accurate predictor for high illness severity in newborns. The aim of this study was to determine the perfusion index values of clinically and hemodynamically stable preterm newborns in the first week of life.

**Methods:**

Perfusion index recordings were performed on the first, third and seventh day of life on 30 preterm newborns. Their state of health was assessed according to clinical and behaviour evaluation and to the Score for Neonatal Acute Physiology.

**Results:**

The median(interquartile range) perfusion index values were 0.9(0.6) on the first, 1.2(1.0) on the third, and 1.3(0.9) on the seventh day, with a significant increase between the first and the third day.

**Conclusions:**

Perfusion index proved to be an easily applicable, non-invasive method for monitoring early postnatal changes in peripheral perfusion. Its trend during the first week of life suggests that its clinical application should take age into account. Further studies are needed to obtain reference perfusion index values from a larger sample of preterm newborns, to identify specific gestational age-related cut-off values for illness and to test the role of perfusion index in monitoring critically ill neonates.

## Background

Newborns admitted to neonatal intensive care units (NICU), and in particular preterm newborns are at high risk for morbidity and mortality during the first week of life because of respiratory distress and bronchopulmonary dysplasia, apnea and bradycardia, necrotizing enterocolitis, intraventricular hemorrhage and periventricular leukomalacia, feeding difficulties, hypoglycemia, hyperbilirubinemia and neonatal sepsis [1]. Most of these neonatal morbidities, and in particular severe neonatal sepsis, which accounts for 11% - 27% of NICU admissions [2-4], are often associated with high mortality rates. Early diagnosis of these neonatal complications represents one of the greatest challenges to neonatologists.

In the latest years, the pulse oximeter has become a vital NICU instrument [5]. The perfusion index (PI), derived from the pulse oximeter signal, has been reported to reflect real-time changes in peripheral blood flow [6,7] and to identify inadequate peripheral perfusion in critically ill newborns. In particular, low PI values have been demonstrated to be an accurate predictor for high illness severity in newborns [8]. The pulse oximeter is an easily applicable, non-invasive diagnostic tool for early screening of perinatal inflammatory diseases such as subclinical chorioamnionitis [9-11]. Reference PI values in newborns have been recently published [12]. However, only few literature data on PI values in preterm neonates are available.

Our aim was to evaluate PI values in a sample of clinically and hemodynamically stable preterm newborns in the first week of life as a prelude to the clinical application of this index.

## Methods

### Patients

Thirty preterm newborns among those admitted to our Neonatal Care Unit (Regina Margherita Children's Hospital, Turin, Italy) were recruited consecutively for this observational study, between November 2007 and April 2008.

The inclusion criteria were clinically and hemodynamically stable conditions, gestational age between 28 and 36 weeks; Apgar score at 1 minute 6 to 10; no need for mechanical ventilation or other invasive procedures at birth.

The patients were considered stable according to the following criteria: normal skin colour, respiratory pattern and cry; normal posture, muscle tone and movements. No need for fraction of inspired oxygen ≥ 0.24, normal heart rate (100-180 beats/min), normal respiratory rate (40-70 breaths/min), absence of prolonged apnoea episodes (>20 sec).

Newborns with risk factors for neonatal sepsis (prolonged or premature rupture of membranes, stained amniotic fluid, presence of maternal infections and/or fever immediately before delivery, chorioamnionitis, very preterm birth, perinatal asphyxia and presence of a central venous catheter) and newborns with congenital malformations (congenital heart diseases, congenital diaphragmatic hernias, neural tube defects) were excluded from the study.

The Institutional Ethics Committee approved the study protocol (Protocol number: 136/CEI). The parents were asked to provide background data with particular attention to pregnancy and delivery, and gave their written informed consent.

### Study design

Clinical examinations, PI measurements and blood analyses were performed in the first week of life in compliance with the following protocol.

During the first 24 hours after admission, clinical and laboratory evaluations were carried out to assess the state of health, and the Score for Neonatal Acute Physiology (SNAP) was calculated [13].

On the first, third and seventh day of life, PI values were recorded by an operator, as well as oxygen saturation (SpO2), pulse rate (PR) and respiratory rate (RR). PI measurement was assessed for 5 min/day in the morning, before feeding, while asleep or quietly awake and far from i.v. treatment. Because ambient light could affect oximeter operation, the probes were wrapped in opaque material. Body temperature and arterial blood pressure (BP) were also measured. Clinical examination was performed to evaluate vigilance, reactivity, crying pattern, skin colour, muscle tone and reflexes, bregmatic fontanelle. The study protocol also stated that, in case of any clinical sign and/or symptom suggesting cardiac pathologies, patients should undergo a cardiological examination and echocardiography, and that they should be excluded from the study if necessary. The presence of vomit and diarrhoea was also evaluated according to Nurse reports. On the first and seventh day of life, a blood sample was collected after PI recording to evaluate gas analysis, haemachrome, creatinine, blood urea, glucose, indirect and direct bilirubin, electrolytes and C-reactive protein.

### Techniques

PI measurement was assessed with VitaGuard VG3100 monitoring system (Getemed Medizin- und Informationstechnik AG, Teltow, Germany) based on Masimo signal extraction technology (SET) (Masimo Corp., Irvine, CA, USA) with the SpO2 sensor placed randomly on either foot and three cardiac electrodes on the chest.

In pulse oximeter, a constant amount of light is absorbed by skin, other tissues, and non-pulsatile blood, whereas a variable amount of light is absorbed by pulsating arterial inflow. PI is a scalar value derived from the magnitude of the pulsations displayed on the plethysmographic waveform. It is calculated as the ratio of the pulsatile component to the non-pulsatile component of the infrared signal returning from the monitoring site, and it reflects the ratio of the pulsatile to the non-pulsatile component of the bloodstream [10]. For Masimo Radical SET the PI upper and lower limits reported by the manufacturer are 0.02-20.00%.

The recorded pulse oximeter signals were stored in a personal computer and analysed through the VitaWin 3 software (Getemed Medizin- und Informationstechnik AG, Teltow, Germany). Only the parts of the waveform where the recorded signal resulted to be artefact-free, and where oximeter-derived heart rate corresponded to cardiac electrode-derived heart rate, were considered valid for statistical analysis. The median PI for each measurement was obtained from the average of PI values recorded at 6-second intervals.

Systolic and diastolic BP were measured indirectly using an oscillometric device (Passport 2, Datascope Corp., MahWah, NJ, USA).

### Statistical analysis

The primary outcome of this study was to identify the PI values in a sample of clinically and hemodynamically stable preterm newborns during the first week of life.

The sample size was estimated on the basis of the PI values obtained from a previous pilot study on 10 patients satisfying the inclusion criteria for the present study (unpublished data) in order to obtain a statistical power of 0.8, a 95% confidence interval for PI with a precision of 0.3 and an estimated standard deviation of 0.8.

Normality was assessed by the Kolmogorov-Smirnov test and by exploratory data analysis. Data were expressed as mean and standard deviation (SD) or medians and inter-quartile range (IR) as appropriate. Differences between paired samples were evaluated by the Friedman test [14] and by a post hoc multiple comparison analysis using the Least Significant Difference (LSD) method [15].

Ninety-five percent confidence intervals for median PI values were calculated with a bootstrap procedure with 10,000 replications. All statistical calculations were performed using commercially available software: Resampling Procedures Version 1.3 (Department of Psychology, University of Vermont, Burlington, VT) for the bootstrap of the medians and SPSS for Windows, Version 15, (copyright SPSS Inc., Chicago, IL, U.S.A.). All statistical tests were two-tailed and P values < 0.05 were considered significant.

## Results

All the thirty preterm newborns enrolled (14 M, 16F) completed the trial. Table 1 gives an overview of their general characteristics. All patients had a normal SNAP score (score values < 10) within the first 24 h after admission; they were thus classified as low severity illness newborns. Their clinical conditions, body temperature, blood pressure, blood and gas analyses and behaviour were normal in the first day of life and remained stable during all the study period. All patients had a progressive reduction in oxygen requirement. None of the patients showed evidence of cardiac pathologies and/or required blood transfusions or pharmacological treatments with cardiorespiratory or peripheral perfusion effects (i.e. xantine, diuretics, steroids, etc).

**Table 1:** Baseline characteristics of the study population

Study population characteristics	mean (SD)
Gestational age (weeks)	32.5 (2.1)
Weight (g)	1700 (401)
Length (cm)	41.1 (3.3)
Head circumference (cm)	29.9 (1.6)
Apgar score at 1 minute	8 (1)
Apgar score at 5 minutes	9 (1)

**Figure 1:**
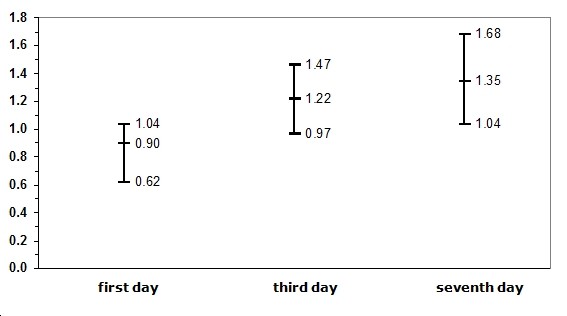
**Median and 95% confidence intervals for median PI values on the first, third and seventh day of life**.

The median PI, PR, SpO2 and BP values were evaluated on day 1, 3 and 7; ninety-five percent confidence intervals for Median PI were illustrated in figure 1.

The PI showed a growth trend with significant weight gain on day 3 and day 7 versus day 1, whereas no significant differences were observed between the third and the seventh day. A similar trend was found comparing the median PR values. There were no significant differences for RR, SpO2 (%) and BP (systolic/diastolic) values during the study period (table 2).

**Table 2:** Median values (inter-quartile range) of PI, PR, RR, SpO2 and BP

Variables	First day	Third day	Seventh day	p value*
PI	0.9 (0.6)	1.2 (1.0)	1.3 (0.9)	<0.001
PR (beats/min.)	126.5 (14.6)	137.4 (20.7)	137.4 (16.7)	0.001
RR (breaths/min.)	42.0 (11.6)	40.1 (6.9)	40.5 (8.8)	0.889
SpO2 (%)	99.7 (1.0)	100.0 (0.6)	99.6 (1.4)	0.078
systolic BP (mmHg)	53.5 (10.0)	55.0 (7.0)	56.5 (14.0)	0.722
diastolic BP (mmHg)	36.0 (11.0)	37.0 (7.0)	38.0 (8.0)	0.845

* Friedman test.

## Discussion

This is the first observational longitudinal study to evaluate PI values during the first week of life in a sample of clinically and hemodynamically stable preterm newborns, with low risk of morbidity.

Our results showed age-related differences in peripheral PI recordings with a significant increase in the median PI value between the first and the third day of life, whereas there was no significant difference between the third and the seventh day.

The peripheral PI trend observed may reflect the physiological variability of the peripheral microvascular blood flow immediately after preterm birth and it could be related to the intrinsic hemodynamic adaptation which occurs in the first days of life. According to this hypothesis, a recent study of systemic blood flow in preterm newborns has pointed out the existence of a perfusion cycle in which low blood flow and high vascular resistance in the first 24 hours are followed by normal-high flow and low resistance, presumably due to vasodilatation [16].

Recently Granelli et al. have published reference values for peripheral PI in a very large sample of healthy newborns between 1 and 120 h of age [12]. Peripheral PI values reported in their study were higher if compared to our data, suggesting that values lower than 0.70 could indicate illness. Our data suggest that physiological PI values in preterm newborns could be lower than in term newborns, thus the cut-off values for PI indicating morbidity should be reconsidered in preterms. Most patients, in fact, (70% on the first measurement, 83% on the second and 90% on the third) presented PI values higher than 0.70, but for some subjects (8, 4 and 2 respectively) PI values were lower than 0.70.

The two studies used different monitoring conditions, which might have affected the results. It is well known that the reliability, accuracy and clinical utility of pulse oximeter remain problematic under certain conditions, such as ambient light exposure, skin pigmentation, dyshemoglobinemia, low peripheral perfusion states and motion artefact [5]. Moreover, during the preliminary phases of our study, we observed that PI recordings were also influenced by the circadian rhythm, time from feeding, contemporary i.v. treatments, jaundice and sleep/wake state.

Pathological conditions such as neonatal sepsis, hypovolaemia and left-to-right shunting congenital heart diseases frequently occur in preterm neonates in the transitional period and can modify microvascular blood flow and cardiovascular adaptation in early neonatal life [17-19]. In order to exclude the presence of these conditions, which could affect peripheral PI, the state of health of our patients was monitored at the enrolment and during the study period through clinical examination, measurement of respiratory and cardiovascular parameters and blood analysis. Moreover, preterm newborns whose gestational age was lower than 28 weeks were not recruited in the present study; this is because very preterm newborns tend to be particularly unstable and often undergo invasive procedures (i.e. mechanical ventilation, CVC, surfactant, PN, etc) which might affect peripheral perfusion.

## Conclusions

Our results suggest that PI is an easily applicable, non-invasive method for monitoring early postnatal changes in peripheral perfusion in preterm infants. The PI trend observed suggests that its clinical application should take subject age and recording conditions into account. Further research on a larger sample of preterm newborns is needed to obtain reference PI values under standardized monitoring conditions, to identify specific gestational age-related cut-off values for illness and to test the role of PI monitoring critically ill neonates.

## List of abbreviations

BP: blood pressure; PI: perfusion index; PR: pulse rate; RR: respiratory rate; SNAP: score for neonatal acute physiology; SpO2: oxygen saturation.

## Competing interests

The authors declare that they have no competing interests.

## Authors' contributions

All the authors have participated in concept, design and revision of the manuscript and they have approved the final version as submitted.
